# Rising to Ostrom’s challenge: an invitation to walk on the bright side of public governance and public service

**DOI:** 10.1080/25741292.2021.1972517

**Published:** 2021-09-03

**Authors:** Scott Douglas, Thomas Schillemans, Paul ‘t Hart, Chris Ansell, Lotte Bøgh Andersen, Matthew Flinders, Brian Head, Donald Moynihan, Tina Nabatchi, Janine O’Flynn, B. Guy Peters, Jos Raadschelders, Alessandro Sancino, Eva Sørensen, Jacob Torfing

**Affiliations:** a Utrecht University, Netherlands; b University of California, Berkeley, CA, USA; c Aarhus University, Denmark; d University of Sheffield, UK; e University of Queensland, Australia; f Georgetown University, USA; g Syracuse University, USA; h University of Melbourne, Australia; i University of Pittsburgh, USA; j Ohio State University, USA; k The Open University Business School; l Roskilde University, Denmark

**Keywords:** Public governance, positive scholarship, policy success, high performance, effectiveness

## Abstract

In this programmatic essay, we argue that public governance scholarship would benefit from developing a self-conscious and cohesive strand of “positive” scholarship, akin to social science subfields like positive psychology, positive organizational studies, and positive evaluation. We call for a program of research devoted to uncovering the factors and mechanisms that enable high performing public policies and public service delivery mechanisms; procedurally and distributively fair processes of tackling societal conflicts; and robust and resilient ways of coping with threats and risks. The core question driving positive public administration scholarship should be: Why is it that particular public policies, programs, organizations, networks, or partnerships manage do much better than others to produce widely valued societal outcomes, and how might knowledge of this be used to advance institutional learning from positives?

## Looking beyond messes and disenchantment

The thickening of transparency and accountability in contemporary democracies (Keane 2009) has led to an enormous amount of energy being directed at pinpointing and dissecting instances in which governments fail our expectations (Flinders 2011; Aleksovska, Schillemans, and Grimmelikhuijsen 2019). By now, there is a vast body of media content, watchdog reports, and scholarly studies on government “disasters” (Hall 1981; Gray and ‘t Hart 1998), “blunders” (King and Crewe 2013; Jennings, Lodge, and Ryan 2018), “failures” (Light 2014; Opperman and Spencer 2016), “blind spots” (Bach and Wegrich 2018), and “fiascos” (Bovens and ‘t Hart 1996). Disappointment and concern are couched in ominous metaphors of “illness,” “breakdown,” “crisis,” “collapse,” “decay,” and even “death” (Diamond 2005; Fukuyama 2014; Levitsky and Ziblatt 2018).

Is this a reflection of underlying realities of declining performance and viability of our public institutions and our systems of government? Or, as the likes of Pinker (2018) and Rosling (2018) would argue, of a culturally entrenched “negativity bias” in the way we look at, judge, and speak about our political and administrative institutions (Vis 2011; Soroka 2014)? There are significant reasons why the latter interpretation is more plausible. We know about the political opportunity structure of bureaucrat-bashing (Schillemans and den Otter 2014). We know about the inclination of citizens, civil servants, and political officeholders to think in stereotypical terms about each other (Raadschelders 2020, 239–243). We know that citizens rate the performance of public sector organizations lower even if they achieve the same results as private sector counterparts (Marvel 2015). We know that some of the disenchantment with government performance is fueled by a lack of understanding and an endemic “expectations gap” (Flinders 2012). We know that even within government, political mindsets and institutional routines are not attuned equally to investigating and learning from successes as they are to anticipating, managing, and taking remedial or reputational action in response to failures and negative performance information (Luetjens and ‘t Hart 2019: George et al. 2020).

What implications might this have for the academic study of public governance? Elinor Ostrom encouraged social scientists to seek out examples of political and resource arrangements that work and then explain them, observing that “an arrangement that works in practice can work in theory” (Fennel 2011) . To be sure, it remains of pivotal importance to generate theoretical explanations for the genesis of failures, stalemates, breakdowns, scandals, and crises remains of pivotal importance. It teaches us what to avoid, mitigate, and stop in the way we set up and run our public institutions. Conversely, however, we must more systematically pursue theoretical explanations that explain instances of solid or even exceptional accomplishments in public governance, to better learn what to embrace, support, and emulate.

As a group of fifteen scholars from different sub-fields, countries, and generations, we argue that public governance research would benefit from launching a self-conscious and cohesive strand of “positive” scholarship, akin to social science subfields like positive psychology (Seligman and Csikszentmihalyi 2000), positive organizational studies (Cameron and Dutton 2003, 4), and positive evaluation (Nielsen, Turksema, and van der Knaap 2015). We call for a program of research devoted to uncovering the factors and mechanisms that enable high performing public problem-solving and public service delivery; procedurally and distributively fair processes of tackling societal conflicts; and robust and resilient ways of coping with threats and risks. The core question driving positive public administration scholarship should be: Why is it that among all the day to day streams of government intervention and public service delivery, particular public policies, programs, organizations, networks, or partnerships manage do much better than others in producing widely valued societal outcomes?

## Building blocks

Walking on the bright side of what governments do and how public governance is performed and how public services are delivered should not follow in the footsteps of business management studies that have become foils for producing “heroic,” agent-centered, rationalistic, success narratives. Instead it should strive to identify micro, meso, and macro *conditions*, and the interplay between agent and institutional context at these levels, i.e. what Little labeled methodological localism (cf. Little 2020, 8, 29), that produce “best of the lot” performances (Meier and Gill 2000). In our effort we shall have to craft usable language, methodologies, and theories to sustain the effort (Compton et al. 2021; Douglas, ‘t Hart, and Van Erp 2021). We must develop conceptualizations of “good governance” that are not just couched in procedural and process terms but also encompass its substantive, material, and psychological impacts. Notice the plural – conceptualisation*s*. Robust debate about “good,” “successful,” or otherwise putatively “positive” governance is not just inevitable but also constitutive of its legitimacy (Mouffe 2000; Flinders 2012; Fung 2012).

As an academic field, we need put in a more systematic and sustained effort to the nature, practices, preconditions, and enablers of highly valuable and widely valued forms of public governance. Fortunately, we do not have to start from scratch. There are significant bodies of case-study and comparative scholarship on policy successes (Bovens, ‘t Hart, and Peters 2001; Marsh and McConnell 2010; Compton and ‘t Hart 2019; Luetjens, Mintrom, and ‘t Hart 2019; Howlett et al. 2022) ; effective government decision making (Crichlow and Schafer 2010); regulatory excellence (Coglianese 2016); public value creation (Moore 1995, 2013; Bryson, Crosby, and Bloomberg 2015; Alford et al. 2017); successful collaborative governance and network management (Dickinson and Sullivan 2014; Emerson and Nabatchi 2015; Page et al. 2015; Cristofoli, Meneguzzo, and Riccucci 2017); American government accomplishments (Light 2002); democratic innovation (Smith 2009; Fung 2012; Hartley, Sørensen, and Torfing 2013; Nabatchi and Leighninger 2015; Sørensen 2017); high-performing and highly reputed public sector organizations (Carpenter 2001; Goodsell 2011; Boin, Fahy, and ‘t Hart 2020); public sector performance improvement regimes (Talbot 2010); unexpected islands of public success in troubled societies (Douglas 2011); exemplary public administrators (Cooper and Wright 1992; Riccucci 1995); institution-building public agency leadership (Boin and Goodin 2007, Boin and Christensen 2008); “pockets of effectiveness” in developing countries (Roll 2014), and resilient (Comfort, Boin, and Demchak 2010) and high-reliability systems handling public tasks in high-risk operating environments (Weick and Sutcliffe 2011). Some of these studies come in clusters where scholars attempt to connect findings – e.g. the relatively cohesive sub-field of performance management (Moynihan et al. 2011; Gerrish 2016) and the recent resurgence of the design approach in public policy analysis (Peters 2018; Peters et al. 2018; Bali, Capano, and Ramesh 2019.

This may feel like a lot. But for this array of disparate studies to evolve into a more cumulative, coherent, publicly visible and practically impactful body of knowledge, we need to drive an integrated agenda for systematic and sustained positive public administration research, and a carefully considered repertoire of impact strategies. This will allow us to be more purposeful and authoritative stimulating the caliber of democratic public governance through positive learning cycles. The remainder of this essay outlines some core themes, conceptual starting points, and methodological considerations that might provide a foundation for a more coherent program of work.

## Core themes

First, a positive public administration program needs to make sense of the discourses of positivity that can be found inpast and contemporary practices of public governance. How do public administrators, political office-holders, citizens, and other stakeholders articulate what they value in the institutions, processes, outputs, and outcomes of public governance? What visions of positive, or good, public administration, public sector performance, and ideals of professional practice are being espoused and transmitted through professional awards, ratings and rankings, and similar mechanisms? What recurrent rhetoric of positive values and success can be discerned and what sense-making functions do they fulfill (Van Assche, Beunen, and Duineveld 2012)?

Second, defying the all too obvious charge that “selection on the dependent variable” is a mortal sin, a positive public administration program should undertake close-up case studies of public organizations, policies, or partnerships that manage to combine high performance with strong legitimacy and adaptive capacity to maintain this constructive equilibrium over considerable periods of time. Such studies can be performed in their own right (e.g. Goodsell 2011; Compton and ‘t Hart 2019; Boin, Fahy, and ‘t Hart 2020), yielding exemplars as well as preliminary insights about enabling conditions, mechanisms and actor repertoires. In addition, and methodologically more elegant, they can be embedded in controlled-comparative designs where they are matched to “negative” cases of program, policy, and organizational failure in otherwise similar conditions (e.g. Bovens, ‘t Hart, and Peters 2001), or identified as “best of the crop” in large-n populations of comparable public programs or organizations (Meier and Gill 2000; Talbot 2010). They also might build on the emerging strand of work on organizational reputation in the public sector, matching its current focus on reputational pressures, blame avoidance, and image repair with studies investigating how reputations in the media and in the public mind relate to the actual performance of those organizations, policies, or partnerships, and whether and how strong performance translates into reputational gains and increased legitimacy (Christensen and Gornitzka 2019).

Finally, numerous accounts in public administration reveal where decision-makers promise to learn from successful policies, organizations, or partnerships in other domains and apply these “best practices” in their own context. But successes are not easily reproduced. Both scholars and administrators should avoid copying and pasting “cookie cutter recipes” and refrain from seeking to simply mimic and transplant “success stories” across time, space, and context (Rose 1993; Marsh and Sharman 2009; Lam and Ostrom 2010, 22). Rather, positive public administration requires a dedicated effort to *learning how to learn* from “what works” in public policy. How dó public actors successfully transfer and circulate programs that “worked” in one context across policy domains, geographies, and temporal contexts (Baker and Walker 2019)? What types of scholarly inputs –journal articles alone won’t hack it – can enhance this learning from others?

## Conceptual starting points

These core questions can be addressed with some shared conceptual starting points. First, positive outcomes can consist both of desirable things happening *and* undesirable things not happening. Research should not only focus on the (antecedents of) positive outcomes but also aim to detect the nonoccurrence of negative events. We should observe fluctuations in the frequency of negative events and positive events equally and perform counterfactual analysis in low-n settings (Ferraro 2009)

Second, as success is multidimensional and multiperspectvist, positive scholars should employ multiple logics in evaluations. We should avoid focusing only on measures for which data is readily available, and not walk away from normative debates about how to assess value (Moore 2013; Mazzucato 2018). The use of multiple evaluative modes to compare, contrast, and weigh the assessments of different stakeholders, constituents, auditors, inspectorates, parliaments, courts, media, and others is important (Marsh and McConnell 2010).

Third, for any positive outcome to remain meaningful, successful governance needs to be robust over time. Private sector studies show that much-heralded companies may go from good to great to gone (Rosenzweig 2014). We should construct time-series through multiple observations of public organizations, policies, and partnerships, gathering assessments across time (Ugyel and O’Flynn 2017).

Finally, we need to be ambitious but humble. Success, like failure, is always the product of a combination of virtu (agency) and fortuna (structure/context). We should avoid romantic explanations and be wary of hero-centric, top-down, episodic explanations of success. It is imperative to explore the role of institutional complexity, organizational learning, adaptive adjustment, bottom-up processes, feedback loops, structured serendipity, and propitious contexts (Evans 1995).

## Methodological considerations

How do we progress from here? What does this mean for public administration research endeavors? As our field is almost uniquely multi-methodological in nature, a positive public administration approach may mean different things in different types of research. Qualitative studies can be immensely valuable in seeking out, observing, and interpreting lived experiences of good governance. Studies will have to move beyond documenting the disgruntlement of frustrated citizens and civil servants, and beyond cozy chats with managers reflecting on their successes. We need to learn from the experiences of those directly involved in and affected by high-performance cultures, well-run public consultations and coproduction processes, effective uses of “right to challenge” provisions, responsive engagements with social entrepreneurs, smart social investment strategies, agile responses to technological turbulence, and resilient coping with major disruptions. We should engage more in appreciative inquiry – including listening to the voices of those making governance work at the coalface and those at the receiving end of policy decisions and service offerings – so we get a better feel of what possible success looks and feels like from the inside (Maynard Moody and Musheno 2003; McQuaid 2019).

Quantitative studies need to identify organizations, policies, or partnerships that are doing markedly better than others, and generate a structured approach for identifying the tangible conditions that explain this success. Important here is addressing negativity biases in research designs. For instance, public administration research on accountability has a very strong focus on pinpointing failures or lacks of accountability, while in comparison, psychological research on accountability has a strong focus on the conditions that make accountability more effective, for instance in attenuating biases, improving compliance, or enriching complex judgments (Aleksovska, Schillemans, and Grimmelikhuijsen 2019). The choice of research questions and theoretical propositions is also crucial. Are they ultimately focused on our ability to produce valued outcomes or to our ability to avoid pitfalls and failure? Positive psychology, for example, champions a refocus on measuring well-being rather than pathology and illness, producing new scales and measurement tools.

Finally, a design focus (van Buuren et al. 2020) can help students of positive public administration understand how different elements of a successful public policy, organization, or partnership hang together and what impacts redesigning and reconstituting them might have. The idea of design induces an appreciation in our research of the craftmanship and continuous tinkering that is necessary to make public governance perform better and adapt over time to maintain high performance in changed circumstances.

On the whole, our methodological considerations center on building a sensitivity to the implicit biases and negative framing of our current methodological apparatus, helping us to challenge “self-evident truths” about slow bureaucracies and failing democratic government (Ostrom 2000) (Table 1).

**Table 1.** Core themes, conceptual starting points and methodological considerations for positive public administration.

Core themes	Conceptual starting points	Methodological considerations
Positivity as frame and discourse Highly valued public policies, organizations, and partnerships Learning to learn from “what works” in public settings	Success can consist of both desirable things happening and undesirable things not happening Success is multidimensional and multiperspectvist Successful governance needs to be robust: both good in time as well as over time Success is likely to be the product of a combination of *virtu* and *fortuna*	Qualitative studies of lived experience Quantitative studies of the antecedents of positive outcomes Design research and design thinking

Public administration research has from its inception centered around questions of what governments can do and how they can do this to the benefit of citizens and communities (Wilson 1887). This has inspired many scholars, yet we believe we can further inspire others – in the academy, in the field practice, and in the public at large – with even more dedication and self-consciousness. By terming it *positive public administration* we aim to follow in the footsteps of movements toward positive scholarship in related disciplines and seek to explicate and strengthen our focus on what we should do as in ours: contributing to the quality of democratic government and effective public governance.

We may not always like government, we but we cannot do without it. Pursuing a positive public administration research agenda challenges us to overcome negativity bias in the way people perceive, evaluate, and study government and governance. It aims to give coherence and a new impetus to important but hitherto disparate efforts to conceptualize, reconstruct, interpret, and learn from instances of successful public governance. Heeding Ostrom’s admonishment to provide more theoretical explanations for empirical instances of government arrangements that work, would provide publics and practitioners with robust “usable knowledge” and constructive “critical friendship” that democratic systems of governance need and thrive on (Raadschelders 2019, 2020).

## References

[CIT0001] Aleksovska, Martina, Thomas Schillemans, and Stephan Grimmelikhuijsen. 2019. “Lessons from Five Decades of Experimental and Behavioral Research on Accountability: A Systematic Literature Review.” *Journal of Behavioral Public Administration* 2 (2): 1–18. doi:10.30636/jbpa.22.66.

[CIT0002] Alford, John, Scott Douglas, Karin Geuijen, and Paul ‘t Hart. (Eds.) 2017. “Special Issue: Ventures in Public Value Management.” *Public Management Review* 19 (5): 589–685. doi:10.1080/14719037.2016.1192160.

[CIT0003] Bach, Tobias, and Kai Wegrich. (Eds.) 2018. *The Blind Spots of Public Bureaucracies and the Politics of Non-Coordination*. London: Palgrave.

[CIT0004] Baker, Tom, and Walker, Chris. (Eds.) 2019. *Public Policy Circulation: Arenas, Agents and Actions*. Cheltenham: Elgar.

[CIT0005] Bali, Azad Singh, Giliberto Capano, and M. Ramesh. 2019. “Anticipating and Designing for Policy Effectiveness.” *Policy and Society* 38 (1): 1–13. doi:10.1080/14494035.2019.1579502.

[CIT0006] Boin, Arjen, and Tom Christensen. 2008. “The Development of Public Institutions. Reconsidering the Role of Leadership.” *Administration and Society* 40 (3): 271–297. doi:10.1177/0095399707313700.

[CIT0007] Boin, Arjen, and Robert Goodin. 2007. “Institutionalizing Upstarts: The Demons of Domestication and the Benefits of Recalcitrance.” *Acta Politica* 42 (1): 40–57. doi:10.1057/palgrave.ap.5500168.

[CIT0008] Boin, Arjen, Fahy, Lauren, and ‘t Hart, Paul. (Eds.) 2020. *Guardians of Public Value: How Public Organisations Become and Remain Institutios*. London: Palgrave Macmillan.

[CIT0009] Bovaird, Tony, and Elke Löffler. 2009. “More Quality through Competitive Quality Awards? An Impact Assessment Framework.” *International Review of Administrative Sciences* 75 (3): 383–401. doi:10.1177/0020852309337687.

[CIT0010] Bovens, Mark, and Paul ‘t Hart. 1996. *Understanding Policy Fiascoes*. New Brunswick: Transaction.

[CIT0011] Bovens, Mark, Paul ‘t Hart, and Guy B. Peters. (Eds.). 2001. *Success and Failure in Public Governance*. Cheltenham: Elgar.

[CIT0012] Bryson, John, Barbara Crosby, and Laura Bloomberg. (Eds.). 2015. *Public Value and Public Administration*. Washington: Georgetown University Press.

[CIT0013] Cameron, Kim S., and Jane E. Dutton. (Eds.). 2003. *Positive Organizational Scholarship: Foundations of a New Discipline*. San Francisco: Berrett-Koehler Publishers.

[CIT0014] Carpenter, Daniel. 2001. *The Forging of Bureaucratic Autonomy: Reputations, Networks and Policy Innovation in Executive Agencies, 1862-1928*. Princeton: Princeton University Press.

[CIT0015] Christensen, Tom, and Åse Gornitzka. 2019. “Reputation Management in Public Agencies: The Relevance of Time, Sector, Audience, and Tasks.” *Administration and Society* 51 (6): 885–914. doi:10.1177/0095399718771387.

[CIT3333] Coglianese, Cary. 2016. *Achieving Regulatory Excellence*. Washington: Brookings.

[CIT0016] Comfort, Louise K., Boin, Arjen, and Demchak, Chris. (Eds.). 2010. *Designing Resilience: Preparing for Extreme Events*. Pittsburgh: Pittsburgh University Press.

[CIT0017] Compton, Mallory, and Paul ‘t Hart. (Eds.). 2019. *Great Policy Successes*. Oxford: Oxford University Press.

[CIT0018] Compton, Mallory, Scott Douglas, Lauren Fahy, Joannah Luetjens, Paul ‘t Hart, and Judith Van Erp. 2021. “Walk on the Bright Side—What Might we Learn about Public Governance by Studying Its Achievements?” *Public Money and Management* (forthcoming).

[CIT0019] Cooper, Terry L., and N. Dale. Wright. (Eds.). 1992. *Exemplary Public Administrators*. San Francisco: Jossey-Bass.

[CIT0020] Crichlow, Scott, and Mark Schafer. 2010. *Groupthink versus High-Quality Decision Making in International Relations*. New York: Colombia University Press.

[CIT0021] Cristofoli, Daniela, Marco Meneguzzo, and Norma Riccucci. (Eds.). 2017. “Special Issue – Collaborative Administration: The Management of Successful Networks.” *Public Management Review* 19 (3): 275–380. doi:10.1080/14719037.2016.1209236.

[CIT0022] Diamond, Jarred. 2005. *Collapse: How Societies Choose to Fail or Succeed*. New York: Viking.

[CIT0023] Dickinson, Helen, and Helen Sullivan. 2014. “Towards a General Theory of Collaborative Performance.” *Public Administration* 92 (1): 161–177. doi:10.1111/padm.12048.

[CIT0024] Douglas, Scott. 2011. “Success Nonetheless, Making Public Utilities Work in Small-scale Democracies Despite Difficult Capital Conditions.” Thesis. Oxford, University of Oxford.

[CIT0025] Douglas, Scott, Paul ‘t Hart, and Judith Van Erp. 2021. Identifying and interpreting governance successes: An assessment tool for classroom use. Ms under review.

[CIT0026] Emerson, Kirk, and Tina Nabatchi. 2015. *Collaborative Governance Regimes*. Washington, DC: Georgetown University Press.

[CIT0027] Evans, Richard. 1995. *Embedded Autonomy: States and Industrial Transformation*. Princeton: Princeton University Press.

[CIT0028] Ferraro, Paul J. 2009. “Counterfactual Thinking and Impact Evaluation in Environmental Policy.” *New Directions for Evaluation* 2009 (122): 75–84. doi:10.1002/ev.297.

[CIT0029] Flinders, Matthew. 2011. “Daring to be a Daniel: The Pathology of Politicized Accountability in a Monitory Democracy.” *Administration and Society* 43 (5): 1–25. doi:10.1177/0095399711403899.

[CIT0030] Flinders, Matthew. 2012. *Defending Politics: Why Democracy Matters in the Early 21st Century*. Oxford: Oxford University Press.

[CIT1111] Fennell, Lee Anne. 2011. Ostrom's Law: Property Rights in the Commons. *International Journal of the Commons*, 5 (1), 9–27.

[CIT0031] French, Richard D. 2019. “Is It Time to Give up on Evidence-Based Policy? Four Answers.” *Policy and Politics* 47 (1): 151–168. doi:10.1332/030557318X15333033508220.

[CIT0032] Fukuyama, Francis. 2014. *Political Order and Political Decay*. Stanford: Stanford University Press.

[CIT0033] Fung, Archon. 2012. “Continuous Institutional Innovation and the Pragmatic Conception of Democracy.” *Polity* 44 (4): 609–624. doi:10.1057/pol.2012.17.

[CIT0034] George, Bert, Martin Baekgaard, Adelien Decramer, Mieke Audenaert, and Stijn Goeminne. 2020. “Institutional Isomorphism, Negativity Bias and Performance Information Use by Politicians: A Survey Experiment.” *Public Administration* 98 (1): 14–28. doi:10.1111/padm.12390.

[CIT0035] Gerrish, Ed. 2016. “The Impact of Performance Management on Performance in Public Organizations: A Meta‐Analysis.” *Public Administration Review* 76 (1): 48–66. doi:10.1111/puar.12433.

[CIT0036] Goodsell, Charles. 2011. *Mission Mystique: Belief Systems in Public Agencies*. Washington: CQ Press.

[CIT0037] Gray, Pat, and ‘t Hart, Paul. (Eds.). 1998. *Public Policy Disasters in Europe: A Comparative Analysis*. London: Routledge.

[CIT0038] Hall, Peter. 1981. *Great Planning Disasters*. London: Penguin.

[CIT0039] Hartley, Jean, Eva Sørensen, and Jacob Torfing. 2013. “Collaborative Innovation: A Viable Alternative to Market Competition and Organizational Entrepreneurship.” *Public Administration Review* 73 (6): 821–830. doi:10.1111/puar.12136.

[CIT0040] Head, Brian. 2010. “Public Management Research: Towards Relevance.” *Public Management Review* 12 (5): 571–585. doi:10.1080/14719031003633987.

[CIT2222] Howlett, Michael, Evert Lindquist, Grace Skogstad, Geneviève Tellier, and Paul't Hart (Eds.) 2022. *Public Policy Success: Lessons from Canadian Experiences*. Oxford: Oxford University Press.

[CIT0041] Jennings, Will, Martin Lodge, and Matt Ryan. 2018. “Comparing Blunders in Government.” *European Journal of Political Research* 57 (1): 238–258. doi:10.1111/1475-6765.12230.

[CIT0042] Keane, John. 2009. *Life and Death of Democracy*. New York: Simon and Schuster.

[CIT0043] King, Anthony, and Ivor Crewe. 2013. *The Blunders of Our Governments*. London: Oneworld.

[CIT0044] Lam, Wai Fung, and Elinor Ostrom. 2010. “Analyzing the Dynamic Complexity of Development Interventions: Lessons from an Irrigation Experiment in Nepal.” *Policy Sciences* 43 (1): 1–25. doi:10.1007/s11077-009-9082-6.

[CIT0045] Levitsky, Steven, and Daniel Ziblatt. 2018. *How Democracies Die*. New York: Penguin Viking.

[CIT0046] Light, Paul. 2002. *Government’s Greatest Achievements: From Civil Rights to Homeland Defense*. New York: Brookings Institution Press.

[CIT0047] Light, Paul. 2014. *A Cascade of Failures. Why Government Fails, and How to Stop It*. Washington: Brookings Center for Effective Public Management.

[CIT0048] Little, Daniel. 2020. *A New Social Ontology of Government. Consent, Coordination, and Authority*. Cam, SU: Palgrave Macmillan.

[CIT0049] Luetjens, Joanne, Michael Mintrom, and Paul ‘t Hart. (eds). 2019. *Successful Public Policy: Lessons from Australia and New Zealand*. Canberra: ANU Press.

[CIT0050] Luetjens, Joanne, and Paul ‘t Hart. 2019. “Governing by Looking Back: Learning From Successes and Failures. An ANZSOG research paper for the Australian Public Service Review Panel”. https://www.apsreview.gov.au/sites/default/files/resources/appendix-c-governing-looking-back.pdf

[CIT0051] Marsh, David, and Allan McConnell. 2010. “Towards a Framework for Establishing Policy Success.” *Public Administration* 88 (2): 564–583. doi:10.1111/j.1467-9299.2009.01803.x.

[CIT0052] Marsh, David, and Jason Sharman. 2009. “Policy Diffusion and Policy Transfer.” *Policy Studies* 30 (3): 269–288. doi:10.1080/01442870902863851.

[CIT0053] Marvel, John D. 2015. “Public Opinion and Public Sector Performance: Are Individuals’ Beliefs about Performance Evidence-Based or the Product of anti–Public Sector Bias?” *International Public Management Journal* 18 (2): 209–227. doi:10.1080/10967494.2014.996627.

[CIT0054] Maynard Moody, Steven, and Michael Musheno. 2003. *Cops, Teachers and Counselors: Stories from the Front Line of Public Service*. Ann Arbor: University of Michigan Press.

[CIT0055] Mazzucato, Mariana. 2018. *The Value of Everything*. London: Allen Lane.

[CIT0056] McQuaid, Michelle. 2019. “How Do A Appreciate Inquiry Summits Help Organizational Systems to Flourish?” *Enschede*. PhD Dissertation, University of Twente.

[CIT0057] Meier, K. J., and J. Gill. 2000. *What Works: A New Approach to Program and Policy Analysis*. New York: Routledge.

[CIT0058] Meier, K. J., L. J. O’Toole, Jr, G. A. Boyne, and R. M. Walker. 2006. “Strategic Management and the Performance of Public Organizations: Testing Venerable Ideas against Recent Theories.” *Journal of Public Administration Research and Theory* 17 (3): 357–377. doi:10.1093/jopart/mul017.

[CIT0059] Moore, Mark. 1995. *Creating Public Value: Strategic Management in Government*. Cambridge, Mass: Harvard University Press.

[CIT0060] Moore, Mark. 2013. *Recognizing Public Value*. Cambridge, Mass: Harvard University Press.

[CIT0061] Mouffe, Chantal. 2000. *The Democratic Paradox*. London: Verso.

[CIT0062] Moynihan, Donald P., Sergio Fernandez, Soonhee Kim, Kelly M. LeRoux, Suzanne J. Piotrowski, Bradley E. Wright, and Kaifen Yang. 2011. “Performance Regimes Amidst Governance Complexity.” *Journal of Public Administration Research and Theory* 21 (Supplement 1): i141–i155. doi:10.1093/jopart/muq059.

[CIT0063] Nabatchi, Tina, and Matt Leighninger. 2015. *Public Participation for 21st Century Democracy*. San Francisco, CA: Jossey Bass.

[CIT0064] Nielsen, Bohni S., Rudi Turksema, and Peter van der Knaap. (Eds.). 2015. *Success in Evaluation: Focusing on the Positives*. New Brunswick: Transaction.

[CIT0065] Opperman, Kai, and Alexander Spencer. (Eds.). 2016. “Fiascos in Public Policy and Foreign Policy: Special Issue.” *Journal of European Public Policy* 23 (5): 643–787.

[CIT5555] Ostrom, Elinor. 2000. “Collective Action and the Evolution of Social Norms.” *Journal of Economic Perspectives* 14 (3): 137–158.

[CIT0066] Page, Stephen, Melissa Stone, John Bryson, and Barbara Crosby. 2015. “Public Value Creation by Cross-Sector Collaborations: A Framework and Challenges of Assessment.” *Public Administration* 93 (3): 715–732. doi:10.1111/padm.12161.

[CIT0067] Peters, B. Guy. 2018. *Policy Problems and Policy Design*. Cheltenham: Edward Elgar.

[CIT0068] Peters, B. Guy, Giliberto Capano, Michael Howlett, Ishander Mukherjee, Meng-Hsuan Chou, and Pauline Ravinet. 2018. *Designing for Policy Effectiveness: Defining and Understanding a Concept*. Cambridge: Cambridge University Press.

[CIT0069] Pinker, Steven. 2018. *Enlightenment Now*. London: Viking.

[CIT0070] Raadschelders, Jos C. N. 2019. “The State of Theory in the Study of Public Administration in the USA: Balancing Evidence-Based, Usable Knowledge and Conceptual Understanding.” *Administrative Theory and Praxis* 41 (1): 3–30. doi:10.1080/10841806.2018.1526517.

[CIT0071] Raadschelders, Jos C. N. 2020. *The Three Ages of Government. From the Person, to the Group, to the World*. Ann Arbor: University of Michigan Press.

[CIT0072] Riccucci, Norma. 1995. *Unsung Heroes: Federal Execucrats Making a Difference*. Washington: Georgetown University Press.

[CIT0073] Roll, Michael. (Ed.). 2014. *The Politics of Public Sector Performance: Pockets of Effectiveness in Developing Countries*. London: Routledge.

[CIT0074] Rose, Richard. 1993. *Lesson-Drawing in Public Policy: A Guide to Learning across Time and Space*. Chatham, NJ: Chatham House.

[CIT0075] Rosenzweig, Phil. 2014. *The Halo Effect: … and the Eight Other Business Delusions that Deceive Managers*. New York: Simon and Schuster.

[CIT0076] Rosling, Hans. 2018. *Factfulness: Ten Reasons We’re Wrong about the World—And Why Things Are Better than You Think*. New York: Flatiron Books.

[CIT0077] Schillemans, Thomas, and Paul den Otter. 2014. “Groeiend Ongemak. Bestuurderspartijen en de Constructie Van Het Vertrouwen in de Overheid.” *Bestuurskunde* 23 (2): 61–70. doi:10.5553/Bk/092733872014023002008.

[CIT0078] Seligman, Martin, and Mikhael Csikszentmihalyi. 2000. “Positive psychology. An introduction.” *The American Psychologist* 55 (1): 5–14. doi:10.1037//0003-066x.55.1.5. 11392865

[CIT0079] Smith, Graham. 2009. *Democratic Innovations: Designing Institutions for Citizen Participation*. Cambridge: Cambridge University Press.

[CIT0080] Sørensen, Eva. 2017. “Political Innovations: Innovations in Political Institutions, Processes and Outputs.” *Public Management Review* 19 (1): 1–19. doi:10.1080/14719037.2016.1200661.

[CIT0081] Soroka, Stuart N. 2014. *Negativity in Democratic Politics: Causes and Consquences*. New York: Cambridge University Press.

[CIT0082] Talbot, C. 2010. *Theories of Performance: Organizational and Service Improvement in the Public Domain*. Oxford: Oxford University Press.

[CIT0083] Ugyel, Lhawang, and Janine O’Flynn. 2017. “Measuring Policy Success: Evaluating Public Sector Reform in Bhutan.” *International Journal of Public Administration* 40 (2): 115–125. doi:10.1080/01900692.2015.1076466.

[CIT0084] Van Assche, Kristoff, Raoul Beunen, and Martijn Duineveld. 2012. “Performing Success and Failure in Governance: Dutch Planning Experiences.” *Public Administration* 90 (3): 567–581. doi:10.1111/j.1467-9299.2011.01972.x.

[CIT0085] van Buuren, Arwin, Jenny M. Lewis, B. Guy Peters, and William Voorberg. 2020. “Improving Public Policy and Administration: The Potential of Design.” *Policy and Politics* 48 (1): 3–19. doi:10.1332/030557319X15579230420063.

[CIT0086] Vis, Barbara. 2011. “Prospect Theory and Political Decision Making.” *Political Studies Review* 9 (3): 334–343. doi:10.1111/j.1478-9302.2011.00238.x.

[CIT4444] Weick, Karl, and Lisa Sutcliffe. 2011. *Managing the Unexpected: Resilient Performance in an Age of Complexity*. New York: Wiley.

[CIT0087] Wilson, Woodrow. 1887. “The Study of Administration.” *Political Science Quarterly* 2 (2): 197–222. doi:10.2307/2139277.

